# Supplementary material for: Drug repurposing for aging research using model organisms
Source: Aging Cell. 2017 Jun 16;16(5):1006–15. doi: 10.1111/acel.12626 (PMC5595691; doi:10.1111/acel.12626)
Supplement: Supplementary file 7 — Data S1 Zip‐Archive of all report cards. [file ACEL-16-1006-s007.zip › RC_UN8.pdf]

UN8

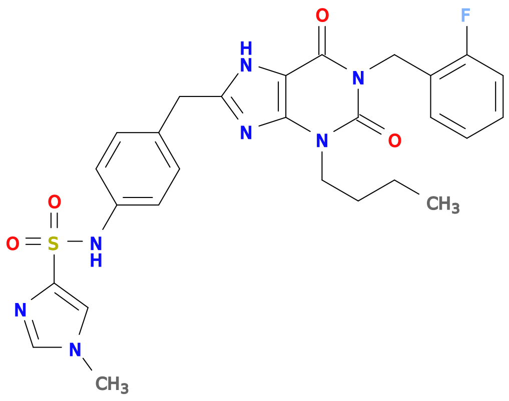

Database identifiers

ChEMBLCompound	CHEMBL245018

Ranking

	Rank	Score
Drosophila	530/697	0.223
C. elegans	562/591	0.014

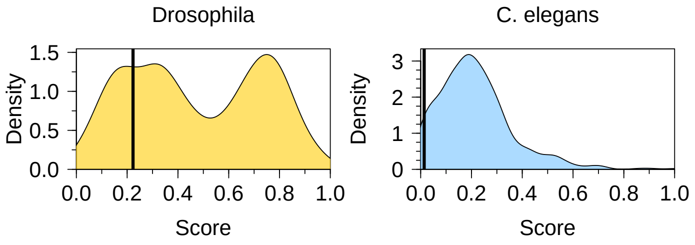

	Ageing implication	Domain conservation	Binding site conservation	Binding affinity	Bioavailability	Lipinski	Promiscuity	Purchasability	Drug approval	**Total**
Drosophila	0.36	0.953	0.946	0.937	(0.9)	-0.05	-0.0	0.0	0.0	0.223
C. elegans	0.36	0.915	0.991	0.937	0.21	-0.05	-0.0	0.0	0.0	0.014

Names

- N-4-3-butyl-1-(2-fluorobenzyl)-2,6-diketo-7H-purin-8-ylmethylphenyl-1-methyl-imidazole-4-sulfonamide
- N-4-3-butyl-1-(2-fluorophenyl)methyl-2,6-dioxo-7H-purin-8-ylmethylphenyl-1-methylimidazole-4-sulfonamide

Roles

ChEBI entry None has no roles

Status

Approved drug (according to ChEMBL)	No
Number of Rule of 5 violations	1
Binding affinity to original target in log units (RF-Score prediction)	7.71
Burns *C. elegans* bioavailability prediction	-5.84

Compound Target Characteristics

Phosphoenolpyruvate carboxykinase, cytosolic [GTP]

Best gene implication in ageing for this target family came from gene Q9Z2V4 via mapping the annotation from Ensembl ENSMUSG00000027513 via mapping the annotation from EntrezGene 18534 via mapping the annotation from GenAgeModels 0497 annotated in GenAge release 17.

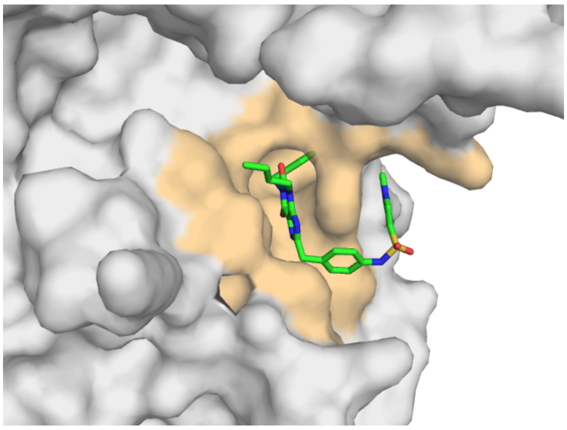

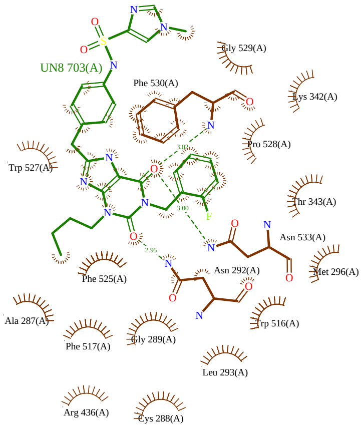

protein	amino acids contacts (binding site)																
PDB:2gmv:chainA:P35558	A	C	G	N	L	M	K	T	R	W	F	F	W	P	G	F	N
sp:P35558:PCKGC_HUMAN	A	C	G	N	L	M	K	T	R	W	F	F	W	P	G	F	N
sp:P07379:PCKGC_RAT	A	C	G	N	L	M	K	T	R	W	F	F	W	P	G	F	N
sp:Q9Z2V4:PCKGC_MOUSE	A	C	G	N	L	M	K	T	R	W	F	F	W	P	G	F	N
tr:A8DYI3:A8DYI3_DROME	A	C	G	N	L	M	A	T	R	W	F	F	W	P	G	F	N
tr:Q7JXB5:Q7JXB5_DROME	A	C	G	N	L	M	A	T	R	W	F	F	W	P	G	F	N
sp:P20007:PCKG_DROME	A	C	G	N	L	L	E	T	R	W	F	F	W	P	G	Y	N
tr:Q8ITY1:Q8ITY1_CAEEL	A	C	G	N	L	L	K	T	R	W	F	F	W	P	G	F	N
tr:Q8ITY2:Q8ITY2_CAEEL	A	C	G	N	L	L	K	T	R	W	F	F	W	P	G	F	N
tr:Q7JKI3:Q7JKI3_CAEEL	A	C	G	N	L	L	K	T	R	W	F	F	W	P	G	F	N
tr:Q8ITY0:Q8ITY0_CAEEL	A	C	G	N	L	L	K	T	R	W	F	F	W	P	G	F	N
tr:Q7JKI2:Q7JKI2_CAEEL	A	C	G	N	L	L	K	T	R	W	F	F	W	P	G	F	N
tr:O02286:O02286_CAEEL	A	C	G	N	L	L	K	T	R	W	F	F	W	P	G	F	N
tr:O44906:O44906_CAEEL	A	C	G	N	L	L	K	T	R	W	F	F	W	P	G	F	N

	whole protein		domain-based		contact-based	
protein	ident	simil	ident	simil	ident	simil
PDB:2gmv:chainA:P35558	0.99	1.0	0.99	1.0	1.0	1.0
sp:P35558:PCKGC_HUMAN	1.0	1.0	1.0	1.0	1.0	1.0
sp:P07379:PCKGC_RAT	0.91	0.98	0.92	0.98	1.0	1.0
sp:Q9Z2V4:PCKGC_MOUSE	0.91	0.98	0.92	0.98	1.0	1.0
tr:A8DYI3:A8DYI3_DROME	0.6	0.8	0.7	0.9	0.94	0.94
tr:Q7JXB5:Q7JXB5_DROME	0.62	0.85	0.7	0.9	0.94	0.94
sp:P20007:PCKG_DROME	0.61	0.84	0.69	0.9	0.82	0.95
tr:Q8ITY1:Q8ITY1_CAEEL	0.32	0.48	0.54	0.81	0.94	0.99
tr:Q8ITY2:Q8ITY2_CAEEL	0.49	0.72	0.57	0.84	0.94	0.99
tr:Q7JKI3:Q7JKI3_CAEEL	0.55	0.81	0.59	0.87	0.94	0.99
tr:Q8ITY0:Q8ITY0_CAEEL	0.49	0.73	0.5	0.71	0.94	0.99
tr:Q7JKI2:Q7JKI2_CAEEL	0.56	0.84	0.59	0.87	0.94	0.99
tr:O02286:O02286_CAEEL	0.54	0.82	0.59	0.87	0.94	0.99
tr:O44906:O44906_CAEEL	0.53	0.8	0.57	0.84	0.94	0.99

Pepck (UniProt:P20007) annotation

Function: Catalyzes the conversion of oxaloacetate (OAA) to phosphoenolpyruvate (PEP), the rate-limiting step in the metabolic pathway that produces glucose from lactate and other precursors derived from the citric acid cycle.
Cofactor: Mn(2+)Note=Binds 1 Mn(2+) ion per subunit. ;
Pathway: Carbohydrate biosynthesis; gluconeogenesis.

Subunit: Monomer.

(Information from UniProt)
